# Surgical Outcomes of Transconjunctival Entropion Repair for Lower Eyelid Involutional Entropion: A Retrospective Case Series

**DOI:** 10.7759/cureus.110403

**Published:** 2026-06-07

**Authors:** Nur Hanisah Mohamad Kani, Niki Ho Wai Wye, Chenshen Lam, Wan Farah Amirah Wan Azlan, Othmaliza Othman

**Affiliations:** 1 Department of Ophthalmology, Faculty of Medicine, Universiti Kebangsaan Malaysia, Kuala Lumpur, MYS; 2 Department of Ophthalmology, Hospital Canselor Tuanku Muhriz, Kuala Lumpur, MYS

**Keywords:** eyelid surgery, involutional entropion, jones technique, lower eyelid retractor, oculoplastic, transconjunctival entropion repair

## Abstract

Background

Involutional entropion is characterized by inward rotation of the lower eyelid due to horizontal lid laxity, eyelid retractor dehiscence, and orbicularis muscle override. Surgical correction is required to address these underlying mechanisms. Transconjunctival entropion repair (TCER) is a comprehensive surgical approach incorporating procedures designed to address the three principal components of involutional entropion.

Method

A retrospective review was conducted of patients with involutional entropion who underwent TCER under local anesthesia. A standardized technique incorporating lateral canthotomy, inferior cantholysis, reattachment of the lower eyelid retractors, preseptal orbicularis myectomy, lateral tarsal strip, and lateral canthopexy was performed. Demographic data, surgical outcomes, recurrence rates, and postoperative complications were analyzed.

Results

A total of 22 eyes from 22 patients were included, with a mean age of 76.3 ± 6.0 years. Immediate postoperative resolution of entropion was achieved in all cases. Overall surgical success was achieved in 19 of 22 eyelids (86.4%). Among patients with long-term follow-up (mean duration: 20.4 ± 16.2 months), surgical success was achieved in 15 of 18 eyelids (83.3%). Recurrence was observed in three eyes at two, three, and 49 months postoperatively. No intraoperative or postoperative complications were observed. No visible external scarring was observed in any patient.

Conclusion

Transconjunctival entropion repair is a comprehensive technique that addresses all underlying mechanisms of involutional entropion, providing good surgical outcomes, a low complication rate, and satisfactory cosmetic results.

## Introduction

Involutional entropion is characterized by the progressive inward rotation of the eyelid margin in the elderly, leading to irritation of the ocular surface. This malposition may result in corneal and conjunctival injury, potentially causing complications such as corneal abrasions, scarring, thinning, neovascularization, and visual impairment, thereby highlighting the clinical importance of timely and effective management [1,2]. The risk increases with age due to a decline in collagen and elastic fibers, accompanied by atrophy of the tarsus and orbital fat [1,3]. The underlying pathogenesis involves a combination of horizontal eyelid laxity, attenuation or disinsertion of the lower eyelid retractors, and overriding of the preseptal orbicularis oculi muscle [4].

Surgical techniques for involutional entropion have evolved to address these underlying anatomical abnormalities. Recognition of lower eyelid retractor dysfunction led to the development of retractor-based corrective procedures [5]. Numerous techniques have since been described, ranging from everting sutures and retractor reinsertion to lateral tarsal strip procedures. However, surgical outcomes vary depending on the mechanisms addressed, with procedures that fail to correct horizontal laxity, retractor dehiscence, and orbicularis override associated with higher recurrence rates [6-9]. Compared to other techniques, transconjunctival entropion repair incorporating lateral canthal support procedures is recognized for effectively addressing the three principal mechanisms of involutional entropion while avoiding a skin incision, offering both functional and cosmetic advantages [10,11].

Despite its advantages, variations in surgical technique and intraoperative identification of the lower eyelid retractors may influence surgical outcomes [12-15]. Optimizing exposure of the tarsal plate and retractor complex may therefore enhance the precision of reattachment and improve surgical stability.

This study aimed to evaluate the clinical outcomes of transconjunctival entropion repair performed at our center using a modified pretarsal pocket technique designed to enhance exposure and facilitate more precise reattachment of the lower eyelid retractors. Outcome measures included surgical success, recurrence, and postoperative complications.

This case series was previously presented as a poster at the 39th Asia Pacific Academy of Ophthalmology Congress (APAO 2024), Bali, Indonesia, in February 2024.

## Materials and methods

Study design and ethics

This retrospective observational case series was conducted at the Oculoplastic Clinic, Hospital Canselor Tuanku Muhriz (HCTM), Kuala Lumpur, Malaysia. Medical records of patients with involutional lower eyelid entropion who underwent transconjunctival entropion repair (TCER) between June 2020 and December 2024 were reviewed.

The study was approved by the Research Ethics Secretariat of Universiti Kebangsaan Malaysia (reference number: UKMPPI/111/8), and adhered to the principles of the Declaration of Helsinki. The requirement for informed consent was waived due to the retrospective nature of the study involving anonymized patient data.

Patient selection and sample size

**Figure 1:**
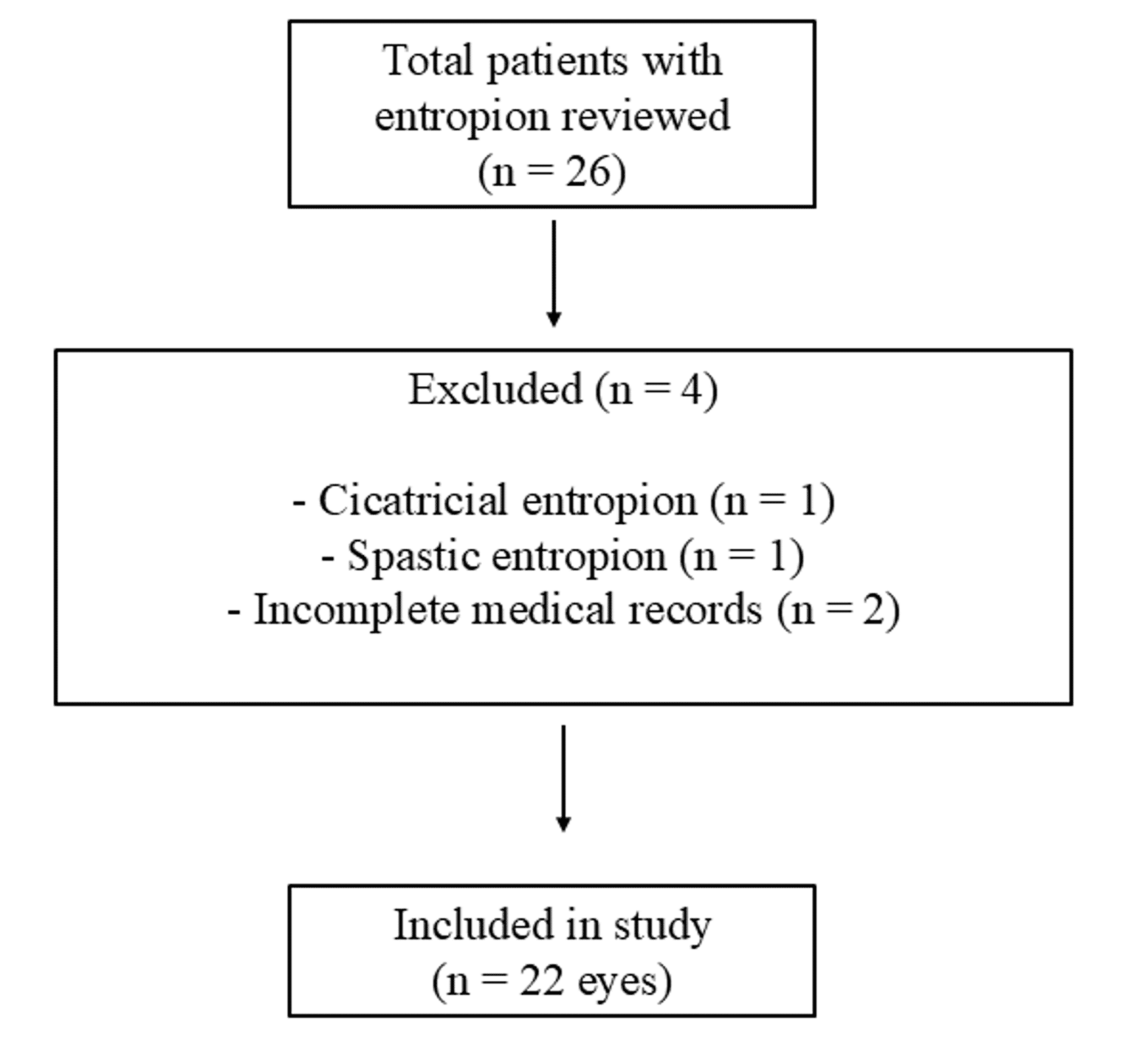
Flowchart of patient selection A total of 26 eyes with entropion were assessed for eligibility, with exclusions applied for cicatricial and spastic entropion and incomplete medical records. The final study cohort comprised 22 eyes. The figure was created using Microsoft PowerPoint (Microsoft Corporation, Redmond, Washington, United states).

Formal sample size calculation was not applicable to this retrospective case series, as all consecutive eligible patients with involutional lower eyelid entropion who underwent TCER during the study period and had complete medical records were reviewed. Exclusion criteria were patients with cicatricial or spastic entropion, previous eyelid surgery affecting lid position, and those with incomplete medical records or less than one month of postoperative follow-up. A total of 26 eyes were screened, and exclusions were applied according to predefined criteria; 22 eyes were included in the final analysis (Figure 1). 

Data collection

Demographic data, including age, gender, and laterality, were collected. Surgical outcomes, including surgical success, recurrence rate, and postoperative complications, were reviewed.

Surgical technique

**Figure 2:**
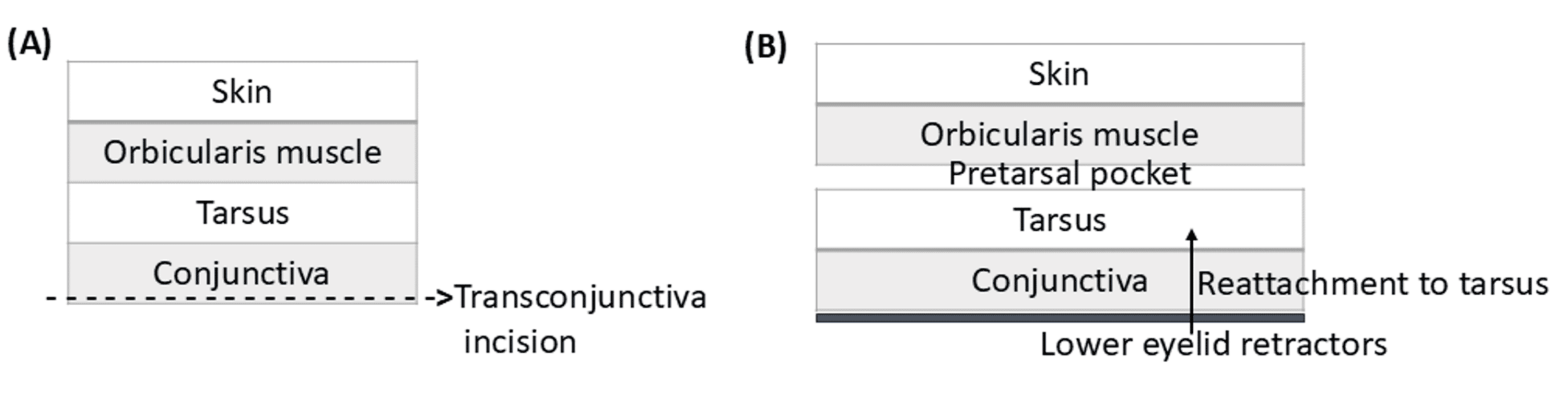
Schematic illustration of transconjunctival entropion repair (A) Transconjunctival incision performed to access the lower eyelid retractors; (B) Creation of a modified pretarsal pocket approximately 1 mm inferior to the tarsal border to improve exposure and facilitate precise lower eyelid retractor reattachment onto the anterior surface of the inferior tarsal plate. Image created by authors using Microsoft PowerPoint (Microsoft Corporation, Redmond, Washington, United States).

**Figure 3:**
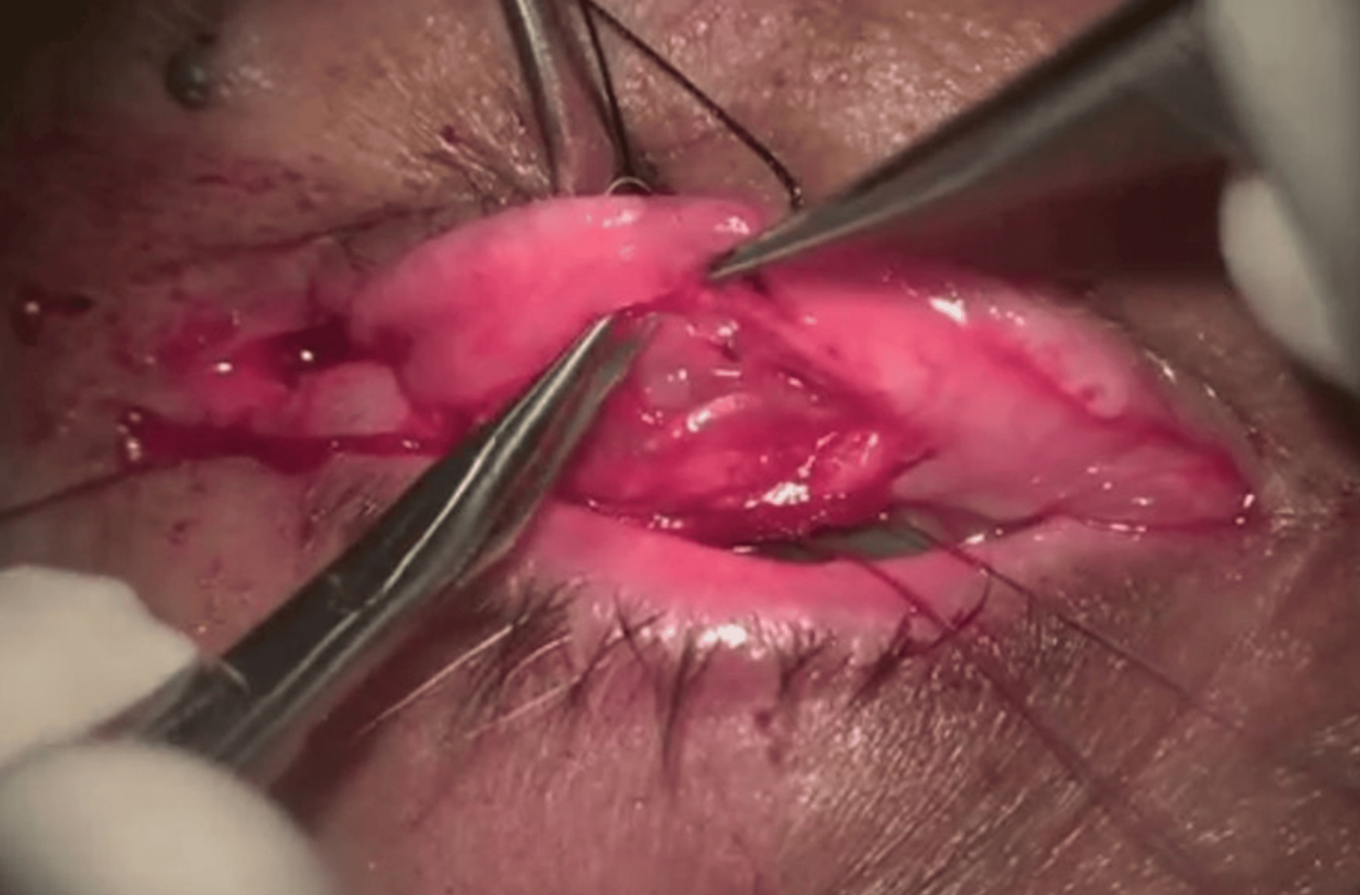
Intraoperative photograph of the modified transconjunctival entropion repair technique Image shows creation of the pretarsal pocket during transconjunctival entropion repair.

All procedures were performed under local anesthesia by an oculoplastic consultant and fellow using a standardized TCER technique based on that described by Dresner and Karesh [10], with modifications as outlined below. This approach was designed to address the three principal pathophysiological components of involutional entropion: horizontal eyelid laxity, lower eyelid retractor dehiscence, and preseptal orbicularis muscle override. The key steps of the modified transconjunctival entropion repair technique are illustrated in Figures 2, 3. 

Anesthesia and Preparation

Local and regional anesthesia with adrenaline was administered to provide adequate analgesia and vasoconstriction. Infiltration was performed at the infraorbital region, lateral canthus, subcutaneous tissue, and subconjunctival plane using 0.5% bupivacaine with adrenaline (1:100,000). A protective ocular conformer was placed over the globe, and a traction suture was applied to the central lower eyelid margin.

Lateral Canthotomy and Inferior Cantholysis

The lateral canthus was clamped with artery forceps for hemostasis, followed by lateral canthotomy using a No. 15 blade and straight tenotomy scissors. A 1-2 cm horizontal incision was made, followed by inferior cantholysis of the inferior crus of the lateral canthal tendon.

Transconjunctival Approach and Retractor Identification

The lower eyelid was everted using a Desmarres retractor. A transconjunctival incision was made approximately 5 mm below the lid margin, just inferior to the tarsal border. Blunt dissection was performed to expose the lower eyelid retractors, identified as a white-gray band beneath the conjunctiva. Identification was facilitated by applying dynamic traction.

Orbicularis Myectomy and Retractor Repair

Three preplaced sutures were inserted into the lower eyelid retractors to aid identification and prevent inadvertent injury. A preseptal orbicularis myectomy was performed to reduce muscle override, with debulking continued until adequate visualization of the underlying structures was achieved. 

A key modification in our technique was the creation of a pretarsal pocket between the orbicularis muscle and the tarsal plate, located approximately 1 mm from the tarsal border. This facilitated exposure of the anterior surface of the inferior one-third of the tarsal plate and allowed precise reattachment of the lower eyelid retractors to their anatomical position. The retractors were plicated using 6-0 polyglactin 910 sutures (Vicryl; Ethicon, Inc., Raritan, New Jersey, United States), restoring vertical tension and correcting the eyelid inversion.

Wound Closure and Correction of Horizontal Eyelid Laxity

The conjunctiva was closed using interrupted buried 8-0 polyglactin 910 sutures. A lateral tarsal strip was fashioned and shortened according to the degree of horizontal laxity. Lateral canthopexy was performed by securing the tarsal strip to the periosteum of the lateral orbital rim using a 5-0 polyester suture (Ethibond; Ethicon, Inc.), with appropriate tension adjustment to achieve optimal eyelid positioning without over-tightening. Closure of the orbicularis and skin at the lateral canthotomy site was performed in layers using 6-0 polyglactin 910 sutures. 

Outcome measures

The primary outcome measures included surgical success and recurrence rate. Surgical success was defined as proper eyelid position without inward rotation at follow-up. Recurrence was defined as the reappearance of inward rotation of the eyelid margin at any postoperative follow-up after initial successful correction. Secondary outcomes included postoperative complications such as overcorrection, ectropion, lid retraction, infection, and corneal complications. Surgical success and cosmetic outcomes were assessed clinically during postoperative follow-up by the treating oculoplastic surgeons based on eyelid position, absence of inward rotation, lash-cornea touch, and overall eyelid appearance.

Statistical analysis

Data were analyzed using descriptive statistics with Microsoft Excel 2021 (Microsoft Corporation, Redmond, Washington, United States). Continuous variables were presented as mean ± standard deviation, while categorical variables were summarized as frequencies and percentages. No comparative or inferential statistical analyses were performed.

## Results

This retrospective review included 22 eyes of 22 patients (mean age: 76.3 ± 6.0 years) who underwent TCER for involutional lower eyelid entropion under local anesthesia. The demographic characteristics at initial presentation are summarized in Table 1.

**Table 1:** Demographic characteristics of patients with involutional entropion who underwent TCER (n = 22) Data are presented as mean ± standard deviation or frequency (percentage). TCER: transconjunctival entropion repair

Demographic Variables	Values
Mean age (years)	76.3 ± 6.0
Age range (years)	66-91
Gender	
Male	14 (63.6%)
Female	8 (36.4%)
Races	
Malay	4 (18.2%)
Chinese	15 (68.2%)
Indian	3 (13.6%)

**Figure 4:**
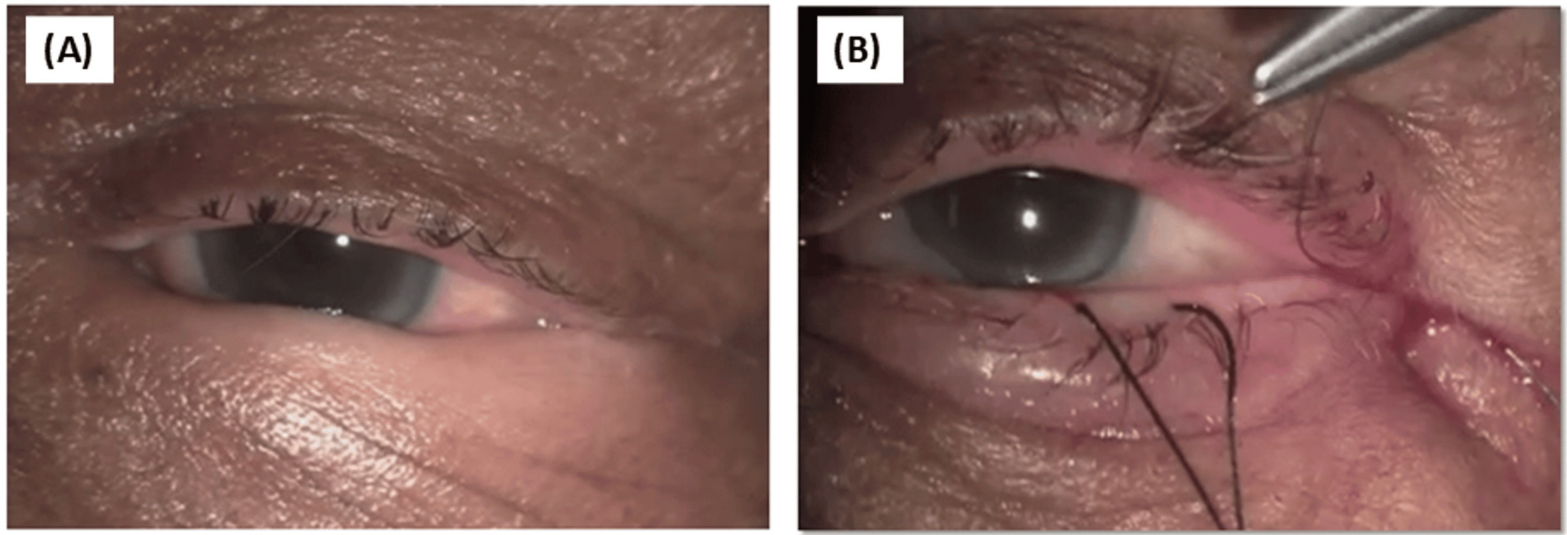
Preoperative and postoperative clinical appearance following transconjunctival entropion repair (A) Preoperative photograph demonstrating lower eyelid involutional entropion with inward rotation of the eyelid margin and lash-cornea touch; (B) Postoperative photograph demonstrating satisfactory anatomical correction with good eyelid contour and no visible external scarring.

All patients underwent unilateral entropion correction, and no intraoperative complications were reported. The mean operative time was 103.8 ± 26.0 minutes. Immediate postoperative resolution of entropion was achieved in all cases (Figure 4).

Long-term follow-up data (mean duration: 20.4 ± 16.2 months) were available for 18 of 22 patients. Overall surgical success was achieved in 19 of 22 eyelids (86.4%). Among patients with long-term follow-up, surgical success was achieved in 15 of 18 eyelids (83.3%). Recurrence was observed in three eyes at two, three, and 49 months postoperatively. Postoperative outcomes, including success and recurrence rates, are summarized in Table 2.

**Table 2:** Postoperative outcomes following TCER ^a^ Overall success among all patients who underwent TCER for involutional entropion; ^b^ Success among patients with long-term follow-up (mean duration: 20.4 ± 16.2 months); ^c^ Recurrences occurred at two, three, and 49 months postoperatively TCER: transconjunctival entropion repair Data are presented as number (percentage). No inferential statistical tests were performed.

Outcome	Total (n = 22 eyelids)	Long-term follow-up (n = 18 eyelids)
Success	19/22 (86.4%) ^a^	15/18 (83.3%) ^b^
Recurrence	3/22 (13.6%) ^c^	3/18 (16.7%)
Postoperative complications	0/22 (0%)	0/18 (0%)

**Figure 5:**
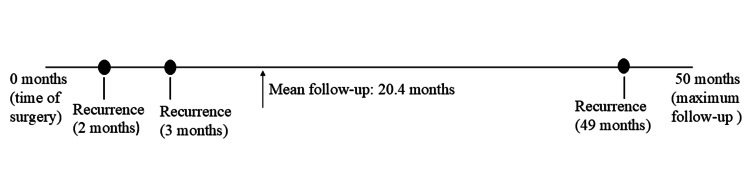
Timeline of postoperative follow-up and recurrence events Recurrence was observed at two, three, and 49 months postoperatively, with a mean follow-up duration of 20.4 months. Created by the authors using Microsoft PowerPoint (Microsoft Corporation, Redmond, Washington, United States).

The timing of recurrence and follow-up duration is illustrated in Figure 5. None of the patients with recurrence opted for revision surgery.

No postoperative complications were observed, including overcorrection, secondary ectropion, lid retraction, scleral show, or lateral canthal dystopia. There were also no cases of corneal epithelial defect or conjunctival edema. All conjunctival incisions healed appropriately within the first postoperative month.

Satisfactory cosmetic outcomes were achieved, with no visible scarring over the lateral canthus, and no cutaneous scar due to the transconjunctival approach.

## Discussion

In this study, TCER incorporating a modified pretarsal pocket technique demonstrated high rates of immediate anatomical correction, with low recurrence and no significant complications. A key modification in our technique is the creation of a pretarsal pocket, which enhances exposure of the anterior tarsal plate and facilitates more precise reattachment of the lower eyelid retractors. Accurate reattachment is critical for restoring vertical eyelid stability, and improved exposure may reduce technical variability and contribute to more durable postoperative outcomes.

TCER is a modification of the Jones technique, based on the principle of lower eyelid retractor plication to restore vertical traction [5]. It employs a minimally invasive transconjunctival approach, allowing direct access to the lower eyelid retractors. The addition of a lateral tarsal strip procedure complements this by addressing horizontal eyelid laxity [16], thereby enabling simultaneous correction of the key pathophysiological mechanisms underlying involutional entropion.

In our retrospective review, all patients demonstrated immediate resolution of entropion following surgery, which remained stable in 19 of 22 eyelids. These findings are comparable to previously reported outcomes of transconjunctival entropion repair. One study reported a success rate of 96.7% (146 of 151 eyelids) over a 12-year period, with a recurrence rate of 3.3% [11]. Similarly, Khan and Meyer reported a 98.2% success rate (112 of 114 eyelids), with no early recurrence and only one late recurrence at 40 months following a modified TCER approach [12]. 

These favourable outcomes are consistent with the principle that successful surgical correction depends on addressing multiple underlying mechanisms. Studies have shown that combining lower eyelid retractor reinsertion with horizontal lid tightening or lateral canthal support is associated with significantly lower recurrence rates, typically ranging from 0% to 5% [10,14]. In contrast, procedures that address only a single pathogenic factor have been associated with higher recurrence rates, as demonstrated by Boboridis et al. [6]. 

Recurrence was observed in three cases at two, three, and 49 months postoperatively. Contributing factors included frequent eye rubbing associated with untreated allergic eye disease, highlighting the importance of addressing concurrent ocular surface conditions such as dry eye and allergy to reduce recurrence risk. Progressive involutional changes may also contribute to late recurrence [15]. Early recurrence in our series may be attributed to technical factors, including inadequate preseptal orbicularis myectomy or insufficient advancement of the lower eyelid retractors. These findings further support the importance of adequately addressing all contributing anatomical components to achieve optimal surgical outcomes [10,14,15].

Although transconjunctival repair has been suggested to increase recurrence due to potential cicatricial changes of the posterior lamella [15], this was not observed in our series. In contrast, more conservative techniques that address only a single pathogenic factor have been associated with higher recurrence rates, reaching up to 17% [6]. In our series, the observed recurrence rates should be interpreted with caution, given the relatively small sample size, in which a small number of events may disproportionately influence percentage-based estimates. Many surgical techniques address only one or two components of the multifactorial pathogenesis of involutional entropion, which may contribute to higher recurrence rates [12]. This concept is summarized in Table 3.

**Table 3:** Pathophysiological mechanisms addressed by common entropion surgical techniques Y, present; X, absent

Surgical Technique	Horizontal lid laxity	Lower Eyelid Retractor Dehiscence	Overriding Preseptal Orbicularis
Transverse everting suture	x	x	Y
Wies Procedure	x	x	Y
Quickert Procedure	Y	x	Y
Transcutaneous Jones Procedure	x	Y	Y
Transconjunctival Entropion Repair	Y	Y	Y

As demonstrated, techniques that fail to simultaneously address horizontal laxity, retractor dysfunction, and orbicularis override may be associated with less favourable outcomes. In contrast, transconjunctival entropion repair allows comprehensive correction of these mechanisms.

No intraoperative or postoperative complications were observed in our series. This is consistent with previous reports demonstrating that the transconjunctival approach, with its limited tissue dissection and concealed incision, is associated with a low risk of complications such as eyelid retraction, scleral show, and secondary ectropion [11,12].

TCER offers several advantages over the transcutaneous approach, including reduced tissue dissection, a concealed incision, and direct access to the lower eyelid retractors [13]. These features contribute to a lower risk of complications such as eyelid retraction, scleral show, and secondary ectropion, while also providing favourable cosmetic outcomes [1]. In contrast, the transcutaneous approach involves more extensive dissection through a skin-muscle flap, which may result in a higher incidence of complications despite potentially comparable recurrence rates [11]. Comparative studies have demonstrated no significant difference in recurrence between the two approaches; however, a higher rate of temporary ectropion has been reported with the transcutaneous technique [17].

Appropriate patient selection remains essential. In particular, assessment of horizontal lid laxity is important, as adequate lateral canthal support is necessary to achieve optimal outcomes and prevent postoperative malposition [18]. In this context, our modified transconjunctival approach aims to optimise surgical precision while maintaining the advantages of a minimally invasive technique.

The demographic characteristics of our cohort were broadly consistent with previous reports, with a mean age of 76.3 years, reflecting the typical elderly population affected by involutional entropion [11,12]. A male predominance was observed in our series, in contrast to prior studies that reported a higher prevalence among females [1,13,19]. This difference may reflect population-specific factors or variations in associated risk factors; however, the underlying reasons remain unclear.

Limitations and future directions

This study is limited by its retrospective design, which is subject to information and selection bias. Follow-up compliance was variable, particularly among elderly patients, and the inclusion of cases with complete follow-up may have introduced selection bias. Data accuracy was dependent on medical records, and no standardized questionnaire was used for data collection. In addition, the relatively small sample size limits the statistical power and generalizability of the findings, and no adjustment for potential confounding variables was performed. The small sample size did not allow meaningful subgroup analysis based on demographic variables such as race.

Future studies incorporating standardized clinical assessments, such as the pinch test, medial distraction test, and lateral distraction test, may allow more objective evaluation of horizontal lid laxity and facilitate tailored surgical planning. Preoperative counselling on avoiding eye rubbing should also be emphasized to reduce recurrence risk. Prospective studies with larger cohorts and longer follow-up, including patient-reported outcomes, would provide further insight into the durability and patient satisfaction associated with this technique.

## Conclusions

TCER represents a comprehensive surgical approach that addresses the multifactorial pathogenesis of involutional entropion. This series demonstrated reliable anatomical correction with low recurrence and no significant complications. The transconjunctival approach offers additional advantages, including avoidance of a cutaneous incision and favorable cosmetic outcomes. Incorporation of a pretarsal pocket technique may further enhance surgical precision by facilitating more accurate reattachment of the lower eyelid retractors.
